# Risk of Diabetes in Older Adults with Co-Occurring Depressive Symptoms and Cardiometabolic Abnormalities: Prospective Analysis from the English Longitudinal Study of Ageing

**DOI:** 10.1371/journal.pone.0155741

**Published:** 2016-05-26

**Authors:** Cassandra Freitas, Sonya Deschênes, Bonnie Au, Kimberley Smith, Norbert Schmitz

**Affiliations:** 1 Department of Epidemiology, Biostatistics and Occupational Health, McGill University, Montreal, Quebec, Canada; 2 Douglas Mental Health University Institute, Montreal, Quebec, Canada; 3 Department of Psychiatry, McGill University, Montreal, Quebec, Canada; 4 Primary Care Research Unit, Sunnybrook Health Sciences Centre, Toronto, Ontario, Canada; 5 Department of Life Sciences, Brunel University London, Uxbridge, Middlesex, England; Providence VA Medical Center and Brown University, UNITED STATES

## Abstract

High depressive symptoms and cardiometabolic abnormalities are independently associated with an increased risk of diabetes. The purpose of this study was to assess the association of co-occurring depressive symptoms and cardiometabolic abnormalities on risk of diabetes in a representative sample of the English population aged 50 years and older. Data were from the English Longitudinal Study of Ageing. The sample comprised of 4454 participants without diabetes at baseline. High depressive symptoms were based on a score of 4 or more on the 8-item binary Centre for Epidemiologic Studies–Depression scale. Cardiometabolic abnormalities were defined as 3 or more cardiometabolic risk factors (hypertension, impaired glycemic control, systemic inflammation, low high-density lipoprotein cholesterol, high triglycerides, and central obesity). Cox proportional hazards regressions assessed the association between co-occurring depressive symptoms and cardiometabolic abnormalities with incidence of diabetes. Multiple imputation by chained equations was performed to account for missing data. Covariates included age, sex, education, income, smoking status, physical activity, alcohol consumption, and cardiovascular comorbidity. The follow-up period consisted of 106 months, during which 193 participants reported a diagnosis of diabetes. Diabetes incidence rates were compared across the following four groups: 1) no or low depressive symptoms and no cardiometabolic abnormalities (reference group, n = 2717); 2) high depressive symptoms only (n = 338); 3) cardiometabolic abnormalities only (n = 1180); and 4) high depressive symptoms and cardiometabolic abnormalities (n = 219). Compared to the reference group, the hazard ratio for diabetes was 1.29 (95% CI 0.63, 2.64) for those with high depressive symptoms only, 3.88 (95% CI 2.77, 5.44) for those with cardiometabolic abnormalities only, and 5.56 (95% CI 3.45, 8.94) for those with both high depressive symptoms and cardiometabolic abnormalities, after adjusting for socio-demographic, lifestyle and clinical variables. These findings suggest that those with high depressive symptoms and cardiometabolic abnormalities are at a particularly increased risk of type 2 diabetes.

## Introduction

Diabetes is one of the most common chronic diseases in nearly every country, with its global disease burden increasing in numbers and significance [1]. According to the International Diabetes Federation (IDF), there are almost 400 million people with diabetes globally, and this number is expected to increase to nearly 600 million by 2035 [2]. The projected increase in prevalence is due in part to increased longevity among people with existing diabetes and also an increase in new cases of diabetes due to population ageing, rising levels of obesity and increased physical inactivity [3].

Type 2 diabetes disproportionally affects older adults [4], a rapidly growing demographic [5]. In England, over one third of the population is comprised of older adults aged 50 years and older [6], 15% of whom have diabetes [7]. Older adults with diabetes are at an increased risk of mortality, microvascular and cardiovascular complications, higher health care costs and utilisation, sleep and appetite disturbances, reduced social and physical functioning, decreased self-care management, reduced quality of life, and cognitive decline [8].

There is increasing interest among researchers to explore the mental health and somatic risk factors for type 2 diabetes, especially among older adults as they are at a particular risk of developing mental disorders and physical illness [9]. Specifically, depression affects approximately 20% of older adults in the United Kingdom [10] and has been shown to be a risk factor for the onset of type 2 diabetes, with recent meta-analyses demonstrating that depression is associated with a 37–60% increase in the risk of type 2 diabetes [11,12]. Diabetes is also strongly influenced by cardiometabolic health. Factors such as hypertension risk, low high-density lipoprotein (HDL) cholesterol, elevated triglycerides, central obesity, impaired glycemic control, and systemic inflammation have been shown to be cardiometabolic risk factors for the development of type 2 diabetes [13–15]. Several studies investigating metabolic dysregulations have done so in the context of the metabolic syndrome, a condition that is comprised of a cluster of general metabolic risk factors for cardiovascular disease and diabetes [14]. One study demonstrated that those with metabolic syndrome have a nearly 5-fold increased risk of developing diabetes than those without metabolic syndrome [16]. Metabolic syndrome is also common among older adults, and as the world population ages, the prevalence is likely to increase [17–19]. Depression has also been found to be associated with the metabolic syndrome, with a recent systematic review suggesting that they are bidirectionally linked [20].

Given that depression and cardiometabolic abnormalities are associated with type 2 diabetes [16,21], individuals with comorbid depression and cardiometabolic abnormalities might be at a higher risk of type 2 diabetes. A recent cross-sectional analysis using data from the English Longitudinal Study of Ageing (ELSA) has shown that individuals with comorbid depression and cardiometabolic abnormalities not only suffer from mental and somatic health issues to a greater extent than individuals with depression without cardiometabolic abnormalities, but are also more likely to exhibit lifestyle and health-related factors associated with poor health outcomes, particularly physical inactivity, poorer self-rated health, lower income and retirement [22]. However, the prospective associations between depression and cardiometabolic abnormalities with incidence of type 2 diabetes are unknown. The aim of the present study was to examine whether individuals with co-occurring high depressive symptoms and cardiometabolic abnormalities are at an increased risk of developing diabetes as compared to those with high depressive symptoms only, those with cardiometabolic abnormalities only and those with no or low depressive symptoms without cardiometabolic abnormalities in a nationally-representative sample of non-institutionalized older adults living in England.

## Methods

### Design/setting and participants

Data for this study come from the English Longitudinal Study of Ageing (ELSA), an ongoing cohort study of a national sample of the English population aged 50 years or older. The original ELSA cohort was drawn from a group of men and women who participated in the Health Survey for England between 1998 and 2001, who were born before March 1, 1952, and who lived in a private household during the first wave of ELSA (2002–2003) [23]. A more detailed description of the ELSA study design is provided elsewhere [23]. For the present study, data from wave 2 (2004–2005) of the ELSA study were used as baseline since this wave was the first to include clinical assessments of blood samples that were collected under fasting conditions by a nurse. Depressive symptoms, cardiometabolic variables, and covariates were all assessed at baseline (Wave 2). Data from wave 3 (2006–2007), wave 4 (2008–2009), wave 5 (2010–2011), and wave 6 (2012–2013) were used to examine the incidence of diabetes (i.e., newly self-reported cases). Participants provided full informed consent to participate in the study and ethical approval was granted by the London Multi-Centre Research Ethics Committee.

### Inclusion Criteria

Inclusion criteria were 1) being between 50 and 80 years of age; 2) having a valid blood sample with a maximum of two missing values for the six cardiometabolic risk factors; 3) having complete data on depressive symptoms; and 4) having attended at least 1 follow-up session after the baseline assessment. Exclusion criteria were proxy respondents and having diabetes at baseline. Specifically, those who responded ‘yes’ to having a physician diagnosis of diabetes at baseline (2004–2005) or those with undiagnosed diabetes, taken as HbA1c levels of 6.5% or above [13], were excluded from the sample.

### Measurements

#### Assessment of Depression

The eight-item binary Center for Epidemiologic Studies–Depression (CES-D) scale was used to assess self-reported depressive symptomology [24,25]. A total summary score was calculated, which could range from 0 to 8. Participants were classified into ‘no or low depressive symptoms’ (total scores of 3 or below) or ‘high depressive symptoms’ (total scores of 4 or more) groups, in accordance with recommendations [26] and previous ELSA studies [22,27,28].

#### Assessment of Cardiometabolic Abnormalities

Six cardiometabolic risk factors were assessed: hypertension risk, low HDL cholesterol, elevated triglycerides, central obesity, impaired glycemic control, and systemic inflammation. The cut-off values for the first 4 of the above mentioned criteria were determined by the criteria for the clinical diagnosis of metabolic syndrome listed in a consensus statement released by the International Diabetes Federation (IDF) [14]. According to the IDF criteria, hypertension risk is defined as having a blood pressure of ≥130/≥85 mmHg or use of anti-hypertensive medication; low HDL cholesterol is defined as <1.0 mmol l^-1^ in men and <1.3 mmol l^-1^ in women; high triglycerides is defined as ≥1.7 mmol l^-1^; and central obesity is defined as having a waist circumference >102 cm in men and >88 cm in women. Impaired glycemic control was defined as having HbA1c levels of ≥6.0% (or ≥42 mmol/mol), as suggested by the International Expert Committee [13,29]; systemic inflammation was defined as having C-reactive protein (CRP) levels >3 mgl^-1^, as suggested by the American Heart Association and the Centres for Disease Control [15].

Participants were categorized as having cardiometabolic abnormalities if they exhibited ≥3 of the above mentioned risk factors. There is no current standardized definition for cardiometabolic groupings; however, previous studies have used cut-offs of ≥2 [30] or ≥3 [31] cardiometabolic abnormalities to identify metabolically unhealthy individuals. We chose to use the more conservative definition of ≥3 cardiometabolic abnormalities, adapted from the IDF criteria for the clinical diagnosis of metabolic syndrome [14], and used in a previous ELSA study [22].

#### Assessment of Diabetes Incidence

Incident diabetes was determined based on self-reported physician-diagnosed diabetes at wave 3 (2006–2007), wave 4 (2008–2009), wave 5 (2010–2011) and wave 6 (2012–2013). The date of diagnosis of diabetes (month/year) was also self-reported. Incident diabetes was epidemiologically classified as type 2 diabetes, in line with previous ELSA studies [22,27,32,33].

#### Covariates

Socio-demographic and lifestyle variables were chosen on the basis of being established risk factors for chronic illness [33]. Demographic variables included age, sex, highest education attained (university degree, less than university degree, no qualification), and income. Income was assessed via quintiles of total non-pension household wealth, which was defined as total household wealth minus household debt and excluding pension savings.

Lifestyle variables included smoking status (never smoker, former smoker, current smoker), frequency of alcohol consumption during the past 12 months (daily, occasional, never), and level of physical activity (sedentary/low, moderate, high). Physical activity was derived from information on occupational and leisure activities classified using the Allied Dunbar Survey of Fitness [34].

Self-reported physician-diagnosed cardiovascular comorbidities including hypertension, angina, congestive heart failure, abnormal heart rhythm, heart murmur, heart attack, and stroke (at least one cardiovascular condition, no cardiovascular condition) was also included in the analysis.

### Statistical Analysis

Four groups were created based on cutoff values chosen *a priori* for CES-D scores (<4 vs ≥4 depressive symptoms) and cardiometabolic abnormalities (<3 vs ≥3 cardiometabolic risk factors): no or low depressive symptoms and no cardiometabolic abnormalities (noDnoCM); no or low depressive symptoms but with cardiometabolic abnormalities (noDCM); high depressive symptoms and no cardiometabolic abnormalities (DnoCM); comorbid high depressive symptoms and cardiometabolic abnormalities (DCM). Analysis of these four groups categorized based on cutoffs of elevated versus low-to-no depressive symptoms and cardiometabolic abnormalities supported by previous research and recommendations, allows for elucidation of the risk of diabetes of a clearly defined comorbid group, comparison groups and a reference group. Sensitivity analyses–described below–explore the risk of diabetes using alternate definitions for these groupings.

The prospective associations between categories of cardiometabolic abnormalities and depressive symptom status with incidence of diabetes were assessed with Kaplan-Meier survival curves and Cox proportional hazards regressions. The regressions were performed to obtain crude and adjusted estimates of diabetes incidence among the four groups. The first model tested the crude association between the four groups and diabetes incidence. The second model adjusted for socio-demographic variables (age, sex, education, income). The third model additionally adjusted for lifestyle variables (level of physical activity, smoking status, alcohol consumption). The fourth model additionally adjusted for cardiovascular comorbidities. The group with no cardiometabolic abnormalities and no or low depressive symptoms served as the reference group. A linear contrast was specified to compare the two depression groups (DCM vs DnoCM).

Multiple imputation by chained equations was used to account for missing covariate data in the Cox proportional hazards analyses [35,36]. Multiple imputation involves the creation of multiple complete datasets by assigning plausible predicted values to otherwise missing values, then analyzes each imputed dataset separately and subsequently combines the point estimates and estimated standard errors to come up with a single, multiply-imputed set of estimates [37]. The exposure, outcome and all the covariate variables were entered into the imputation procedure. Ten imputations were performed. Among the sample, the covariates age, sex, and cardiovascular comorbidity had no missing values. Education (0.02% missing), income (1.53% missing), physical activity (0.04% missing), smoking (0.02% missing), and alcohol consumption (6.92% missing) were imputed as ordinal variables.

For those who reported diabetes, follow-up time was calculated as the time elapsed (in months) between the date of the baseline interview date and the date of the reported diagnosis of diabetes. For those who reported diabetes but did not report the associated date of diagnosis, the follow-up time was calculated as the time elapsed from to the baseline interview date to the midpoint between (a) the last assessment in which they reported not having diabetes and (b) the assessment in which they did report having diabetes. For those who did not report diabetes, follow-up time was calculated as the time elapsed between the date of baseline and most recent interview.

The estimates for risk of diabetes in the Cox regression models are presented as hazard ratios (HRs) and associated 95% confidence intervals (CIs). Statistical tests (Schoenfeld residuals test) and graphical plots (Kaplan-Meier observed survival curves versus Cox predicted curves, and log-log survival plots) were used to confirm the proportional hazards assumption.

To provide additional insight into the interactive effects of depressive symptoms and cardiometabolic abnormalities on diabetes, we tested for the occurrence of a biological interaction. An additive effect exists when two conditions act independently from each other to affect a disease outcome; that is, the risk for those suffering from both conditions equals the sum of the risks for those suffering from only one condition [38]. A biological interaction exists when the risk of the outcome based on having both conditions is in excess of what would be expected under the additive model [39]. Biological interaction (i.e., departure from additivity) was measured using the Synergy index (S) [40,41]. Applied to the hazard model, S was calculated with the formula:

$$S=\frac{\left(HR_{11}-1\right)}{\left(HR_{10}-1)+(HR_{01}-1\right)}$$

Where HR_11_ is the HR for those with high depressive symptoms and cardiometabolic abnormalities, HR_10_ is the HR for those with high depressive symptoms only, and HR_01_ is the HR for those with cardiometabolic abnormalities only. The S can be interpreted as the excess risk from exposure (to both exposures) when there is an interaction relative to the excess risk from exposure (to both exposures) without interaction [41]. An S > 1 implies a greater than additive effect (synergistic effect), whereas S = 1 implies no interaction (exactly additive, no interaction), and an S < 1 implies a less than additive effect (antagonistic interaction). Using multiple imputation by chained equations, a multiply-imputed S and associated 95% confidence interval were generated for all four models.

Several sensitivity analyses were performed. First, complete-case analyses of Cox proportional hazards regressions were performed. The S and associated 95% confidence interval was generated using the bootstrap percentile method [42] which involves generating 2000 bootstrap samples (with replacement) from the complete-case dataset. S was estimated in each of these new samples and the 95% confidence interval was estimated as the 2.5^th^ and 97.5^th^ percentiles of the resulting bootstrap sampling distribution. Second, sensitivity analyses considering both less stringent and more stringent definitions for the cutoff criteria of both the depressive symptoms (≥3 and ≥5 depressive symptoms) and cardiometabolic abnormalities (≥2 and ≥4 cardiometabolic risk factors) groupings were performed. Third, a sensitivity analysis that included those with undiagnosed (HbA1c ≥6.5% or ≥48 mmol/mol) at baseline was conducted. Fourth, in order to determine the effect of each cardiometabolic factor on diabetes incidence, a logistic regression analysis was performed with each cardiometabolic factor entered separately as predictors, adjusted for age, sex, level of education, income, smoking status, level of physical activity and alcohol consumption. Fifth, incidence of diabetes was assessed using both CES-D scores and number of cardiometabolic risk factors as two continuous variables, and their interaction term, adjusting for socio-demographic, lifestyle and clinical variables (multiplicative interaction). All analyses were performed using STATA SE version 13.1 (StataCorp LP, College Station, TX, USA).

## Results

**Fig 1:**
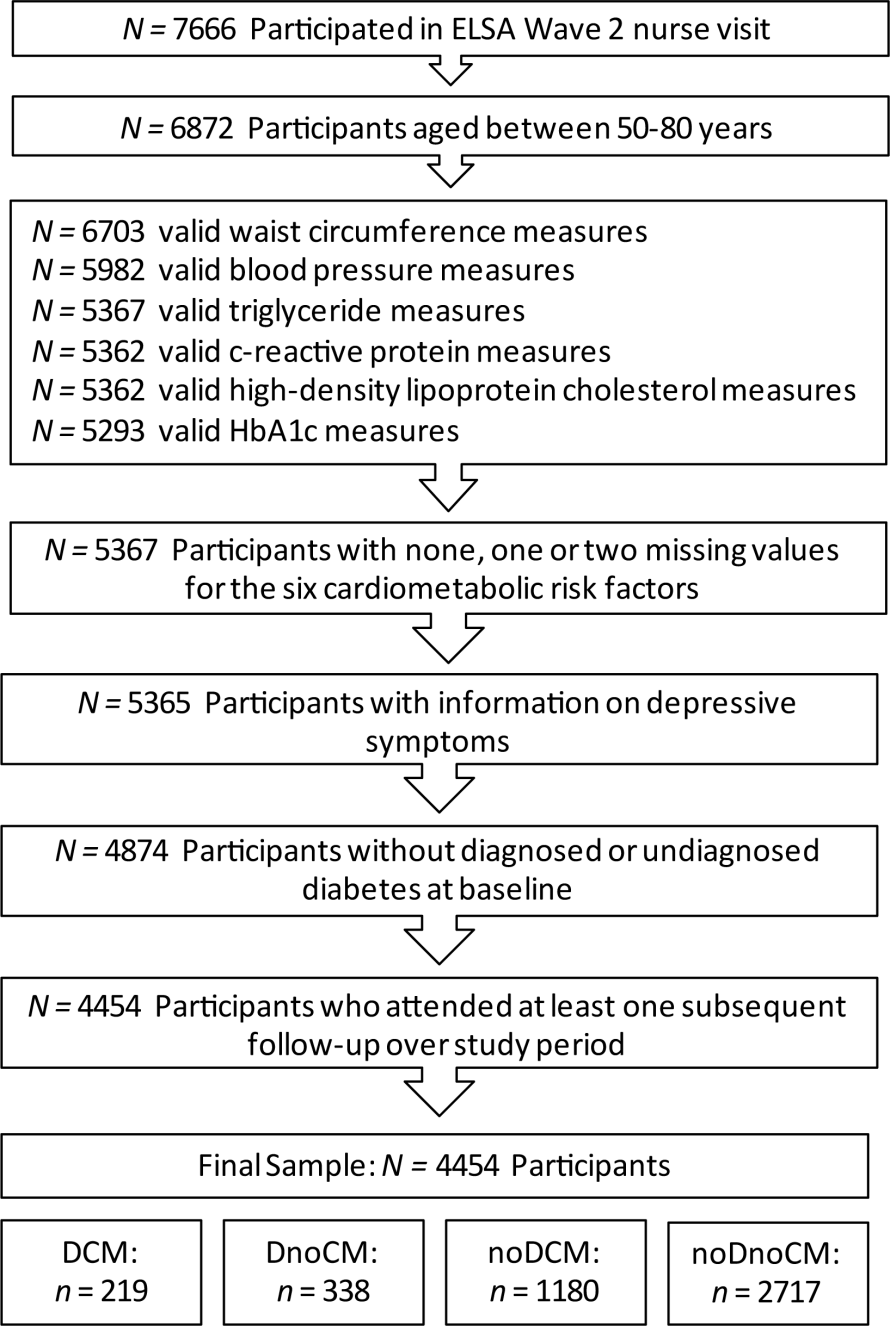
Participant Flow Chart. DCM: comorbid high depressive symptoms and cardiometabolic abnormalities group; DnoCM: high depressive symptoms only group; noDCM: cardiometabolic abnormalities only group; noDnoCM: no or low depressive symptoms and no cardiometabolic abnormalities group; ELSA: English Longitudinal Study of Ageing.

A total of 7666 participants completed a nurse visit at wave 2, of which 6872 participants were between the ages of 50–80 years. Participants with valid waist circumference measures (n = 6703), valid blood pressure measures (n = 5982), valid triglyceride measures (n = 5367), valid CRP measures (n = 5362), valid HDL cholesterol measures (n = 5362) and valid HbA1c measures (n = 5293) were considered. However, only those with two or fewer missing values for any of the 6 cardiometabolic risk factors (n = 5367) and those with CES-D scores (n = 5365) were included. Those with diabetes at baseline (n = 491) were excluded from the analysis. As well, those who did not return for any of the subsequent follow-ups (n = 420) were excluded. The final sample for the present study included 4454 participants (Fig 1).

Compared to those for whom a blood sample was obtained, those who did not obtain a blood sample were more likely to have high depressive symptoms (18% vs 13%), no qualifications (41% vs 34%), be in the lowest wealth quintile (22% vs 16%), be non-alcoholic drinkers (13% vs 9%), be sedentary (9% vs 3%), have a cardiovascular comorbidity (66% vs 50%) and were less likely to be employed (24% vs 35%). Compared to those with 2 or fewer missing values for the cardiometabolic risk factors, those with 3 or more missing values were more likely to have high depressive symptoms (17% vs 13%), have no qualifications (39% vs 34%), be retired (56% vs 49%), be in the lowest wealth quintile (22% vs 16%), be non-alcoholic drinkers (13% vs 9%), be sedentary (8% vs 3%) and have a cardiovascular comorbidity (63% vs 50%). Compared to those who returned for at least one follow-up visit, those who did not return for any subsequent follow-ups were more likely to be male (50% vs 45%), have no qualifications (47% vs 32%), be in the lowest wealth quintile (20% vs 14%), to be smokers (20% vs 15%), to be sedentary (7% vs 2%) and have a cardiovascular comorbidity (51% vs 46%).

There were 219 (4.9%) participants in the DCM group, 338 (7.6%) participants in the DnoCM group, 1180 (26.5%) participants in the noDCM group, and 2717 (61.0%) participants in the noDnoCM group. Characteristics of the sample according to the four depressive symptom and cardiometabolic abnormalities groups are presented in Table 1. Notably, in our sample, those with both high depressive symptoms and cardiometabolic abnormalities were more likely to be less educated, less wealthy, be a non-drinker, less physically active, and have at least one cardiovascular comorbidity, as compared to the other groups.

**Table 1:** Characteristics of the sample according to depression-cardiometabolic groupings.

	noDnoCM	noDCM	DnoCM	DCM	*p*-value*
N (total:4454)	2717	1180	338	219	
AGE (mean,SD)	63.3 (7.52)	64.7 (7.63)	63.8 (8.28)	64.6 (7.93)	*p* < 0.001
SEX (N,%)					
Male	1320 (48.58)	500 (42.37)	102 (30.18)	73 (33.33)	*p* < 0.001
Female	1397 (51.42)	680 (57.63)	236 (69.82)	146 (66.67)	
EDUCATION (N,%)					
University degree or equivalent	464 (17.08)	126 (10.68)	34 (10.06)	15 (6.88)	*p* < 0.001
Less than university	1523 (56.05)	605 (51.27)	176 (52.07)	105 (48.17)	
No qualification	730 (26.87)	449 (38.05)	128 (37.87)	98 (44.95)	
TOTAL NON-PENSION HOUSEHOLD WEALTH QUINTILE (N,%)^a^					
5 (Highest)	762 (28.56)	203 (17.42)	66 (19.70)	22 (10.09)	*p* < 0.001
4	645 (24.18)	269 (23.09)	49 (14.63)	37 (16.97)	
3	529 (19.83)	265 (22.75)	75 (22.39)	55 (25.23)	
2	436 (16.34)	233 (20.00)	73 (21.79)	35 (16.06)	
1 (Lowest)	296 (11.09)	195 (16.74)	72 (21.49)	69 (31.65)	
SMOKING STATUS (N,%)					
Never Smoker	1109 (40.83)	430 (36.44)	110 (32.54)	71 (32.42)	*p* < 0.001
Ex-Smoker	1251 (46.06)	578 (48.98)	155 (45.86)	103 (47.03)	
Current Smoker	356 (13.11)	172 (14.58)	73 (21.60)	45 (20.55)	
ALCOHOL CONSUMPTION (N,%)					
Daily	552 (21.60)	156 (14.21)	42 (13.95)	25 (13.02)	*p* < 0.001
Occasional	1854 (72.56)	841 (76.59)	219 (72.76)	135 (70.31)	
None	149 (5.83)	101 (9.20)	40 (13.29)	32 (16.67)	
PHYSICAL ACTIVITY (N,%)					
Sedentary/Low	427 (15.72)	322 (27.29)	129 (38.17)	90 (41.28)	*p* < 0.001
Moderate	1531 (56.37)	639 (54.15)	157 (46.45)	113 (51.83)	
High	758 (27.91)	219 (18.56)	52 (15.38)	15 (6.88)	
CARDIOVASCULAR COMORBIDITY (N, %)					
Yes	1100 (40.49)	669 (56.69)	161 (47.63)	138 (63.01)	*p* < 0.001

^a^Total non-pension household wealth defined as total household wealth (excluding pension savings) minus household debt.

*Chi-square (ANOVA for age) *p*-values assess the main effect between all four groups. DCM: comorbid high depressive symptoms and cardiometabolic abnormalities group. DnoCM: high depressive symptoms only group. noDCM: cardiometabolic abnormalities only group. noDnoCM: neither high depressive symptoms nor cardiometabolic abnormalities group.

**Fig 2:**
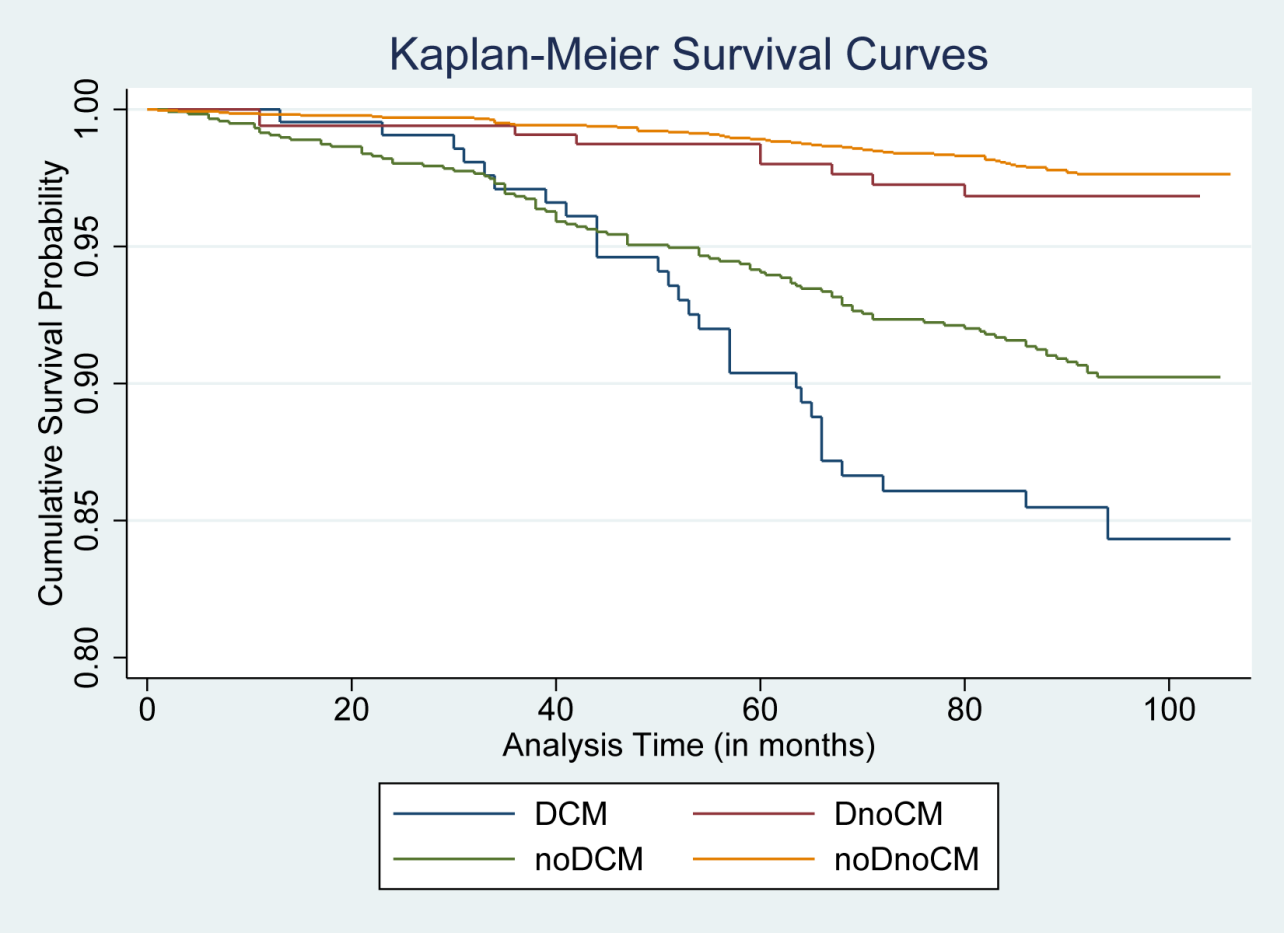
Kaplan-Meier Survival Curves stratified by depression-cardiometabolic groupings. DCM: comorbid high depressive symptoms and cardiometabolic abnormalities group; DnoCM: high depressive symptoms only group; noDCM: cardiometabolic abnormalities only group; noDnoCM: no or low depressive symptoms and no cardiometabolic abnormalities group.

A total of 193 incident cases of diabetes were reported over an average of 81.7 months. Kaplan-Meier survival curves for the incidence of diabetes among the four depression-cardiometabolic groups (Fig 2). Those with high depressive symptoms and cardiometabolic abnormalities demonstrated the lowest cumulative survival probability as compared to the other three groups. The number of people at risk for each group, the number of people lost to follow-up during each time period cutoff of analysis as well as the cumulative survival probability for each time interval are detailed in S1 Table.

Those with high depressive symptoms and cardiometabolic abnormalities had the highest incidence of diabetes with 20.3 per 1000 person-years. Those with cardiometabolic abnormalities only had an incidence rate of 12.8 per 1000 person-years, those with high depressive symptoms only had a rate of 4.1 per 1000 person-years, and those with no or low depressive symptoms and no cardiometabolic abnormalities had a rate of 2.9 per 1000 person-years (Table 2).

**Table 2:** Incidence of diabetes according to depression-cardiometabolic groupings.

	Total	noDnoCM	noDCM	DnoCM	DCM
N	4454	2717	1180	338	219
Incident cases	193	55	100	9	29
Person-Years (PY) follow-up	30316.5	18844.8	7821.3	2218.9	1431.5
Incidence rate (per 1000 PY)		2.9	12.8	4.1	20.3

DCM: comorbid high depressive symptoms and cardiometabolic abnormalities group; DnoCM: high depressive symptoms only group; noDCM: cardiometabolic abnormalities only group; noDnoCM: no or low depressive symptoms and no cardiometabolic abnormalities group.

Table 3 presents the HRs for diabetes across the four groups from the multiple imputation analyses. Those with no or low depressive symptoms and no cardiometabolic abnormalities served as the reference group. After adjusting for socio-demographic, lifestyle, and cardiovascular comorbidities (model 4), the HR was 5.56 (95% CI 3.45, 8.94) for the DCM group, 1.29 (95% CI 0.63, 2.64) for the DnoCM group, and 3.88 (95% CI 2.77, 5.44) for the noDCM group. Contrast analyses suggested a significant difference between the two depression groups (DCM vs DnoCM; p<0.001).

**Table 3:** Hazard Ratios (95% CI) of diabetes across depression-cardiometabolic groupings using multiple imputation by chained equations.

Cox Regression HRs (95% CI)	noDnoCM	noDCM	DnoCM	DCM	S (95% CI)
Model 1: Unadjusted	1.00	4.41 (3.18, 6.13)	1.40 (0.69, 2.83)	7.03 (4.48, 11.02)	
Model 2: Adjusted for age, sex, education, income	1.00	4.11 (2.95, 5.73)	1.36 (0.67, 2.77)	6.16 (3.88, 9.78)	
Model 3: Model 2 + adjusted for physical activity, smoking, alcohol consumption	1.00	4.09 (2.93, 5.72)	1.31 (0.64, 2.68)	5.96 (3.71, 9.56)	
Model 4: Model 3 + adjusted for cardiovascular comorbidity	1.00	3.88 (2.77, 5.44)	1.29 (0.63, 2.64)	5.56 (3.45, 8.94)	1.43 (0.59, 2.28)

DCM: comorbid high depressive symptoms and cardiometabolic abnormalities group; DnoCM: high depressive symptoms only group; noDCM: cardiometabolic abnormalities only group; noDnoCM: no or low depressive symptoms and no cardiometabolic abnormalities group; HR: Hazard Ratio; S: Synergy Index; CI: Confidence Interval.

Testing for a biological interaction revealed an S of 1.43 (95% CI 0.59, 2.28) in the fully adjusted model (model 4). The point estimate of greater than 1 suggests a synergistic interaction between high depressive symptoms and cardiometabolic abnormalities under the additive model. However, because the CIs for these estimates included 1, the interaction was not statistically significant.

There were no significant differences between the results of the multiple imputation analyses and the complete-case analyses, both analyses demonstrated that the comorbid group had the highest risk of developing type 2 diabetes over the follow-up period and both also suggested a positive synergistic interaction that was not statistically significant (S2 Table). Results of the sensitivity analyses performed using the alternate definitions for the cut-off criteria for depressive symptoms and cardiometabolic abnormalities demonstrated that the results were qualitatively unchanged (S3–S6 Tables). In the analysis that included those with undiagnosed diabetes (HbA1c ≥6.5% or ≥48 mmol/mol) at baseline, the results were similar to those of the main analysis. The hazard ratio for diabetes was 7.55 (95% CI 4.80, 11.89) for the DCM group, 1.27 (95% CI 0.60, 2.69) for the DnoCM group, and 5.14 (95% CI 3.70, 7.14) for the noDCM group, with the noDnoCM group as reference, in the fully adjusted model (S7 Table). In the analysis that considered both CES-D score and number of cardiometabolic risk factors as two continuous variables and included their interaction term (multiplicative interaction), the hazard ratio for the CES-D score variable was 1.08 (95% CI 0.87, 1.32), for the cardiometabolic risk factors variable was 2.01 (95% CI 1.72, 2.35) and for their interaction term was 1.01 (95% CI 0.95, 1.07), in the fully adjusted model. The results of all the sensitivity analyses are consistent with those of the main analysis. Assessing the association between each cardiometabolic risk factor and type 2 diabetes adjusting for socio-demographic and lifestyle factors revealed that abnormal HbA1c levels (OR 8.32, 95% CI 5.55, 12.49) was most associated with type 2 diabetes, though elevated triglyceride levels (OR 2.27, 95% CI 1.55, 3.34), central obesity (OR 1.93, 95% CI 1.29, 2.91) and low HDL levels (OR 1.81, 95% CI 1.09, 3.01) were also significantly associated with diabetes. Elevated CRP levels (OR 1.44, 95% CI 0.99, 2.08) and elevated blood pressure (OR 1.36, 95% CI 0.92, 2.01) demonstrated positive but non-statistically significant associations with type 2 diabetes.

## Discussion

The goal of this study was to examine the prospective associations between depressive symptoms and cardiometabolic abnormalities with incidence of diabetes over approximately 8 years of follow-up in a nationally-representative sample of older adults living in England.

Depression and cardiometabolic abnormalities are independent risk factors for diabetes [16,21]. However, our prospective analysis demonstrated that those with co-occurring high depressive symptoms and cardiometabolic abnormalities had the highest risk of developing diabetes, and this association remained after adjusting for socio-demographic, lifestyle and clinical factors. In addition, relative to those with no or low depressive symptoms and no cardiometabolic abnormalities, the risk of diabetes was significantly greater for those with cardiometabolic abnormalities only (i.e., without depression), but not for those with high depressive symptoms only (i.e., without cardiometabolic abnormalities).

Previous research with the ELSA cohort showed that elevated depressive symptoms were associated with an increased risk of diabetes [33]. Recently, another ELSA study demonstrated that those with both elevated depressive symptoms and high CRP levels were also at an increased risk of developing diabetes [27]. Our current study expands on this research by investigating the effect of several cardiometabolic abnormalities, including CRP, on the relationship between depression and diabetes. To our knowledge, this is the first study to examine the longitudinal associations between comorbid high depressive symptoms and cardiometabolic abnormalities with the incidence of diabetes. Overall, we found that those with co-occurring high depressive symptoms and cardiometabolic abnormalities have a higher risk of type 2 diabetes.

While the effect of cardiometabolic risk factors on development of type 2 diabetes is well-established [13,16,43], depression may amplify the effect of these cardiometabolic factors on risk of diabetes. One proposed pathway linking depression with cardiometabolic dysregulation is via its association with obesity [44]. Abdominal adipose tissue produces inflammatory cytokines, including C-reactive protein (CRP), and hormones that contribute to immuno-metabolic responses associated with metabolic disease and depression [45]. As a consequence, these inflammatory factors induce a reduction in high-density lipoprotein (HDL) cholesterol [46], and over time, the effects of chronic inflammation may prompt insulin resistance [47] and resistance to the “satiety hormone” leptin, thus disrupting hunger signals, potentially leading to abdominal fat accumulation [48]. In addition to cardiometabolic dysregulations, depression is also associated with neuroendocrine dysregulations such as hyperactivation of the hypothalamic-pituitary-adrenocortical axis and sympathetic nervous system dysfunction, which have been shown to affect risk factors for type 2 diabetes including abdominal fat accumulation, glucose metabolism and blood pressure regulation [45,49,50].

Due to evidence suggesting that depression seems to be particularly associated with obesity-related cardiometabolic risk factors, Vogelzangs et al [51] proposed that depression and obesity may stimulate each other’s occurrence, but once both are present, multiple progressive metabolic abnormalities may arise that increasingly worsen the depression as well as the metabolic outcome. Over time, this accumulation of metabolic abnormalities may lead to the development of diabetes.

Our results also suggest that depressive symptoms without metabolic abnormalities are not significantly associated with an increased risk of diabetes. Since depression is a heterogeneous condition, it is possible that depression is associated with diabetes particularly when there are comorbid metabolic conditions, however further research is required in order to corroborate our findings.

Our findings suggest that not depression per se but the co-occurrence of high depressive symptoms and cardiometabolic abnormalities might lead to an increased risk of diabetes. However, the pathways linking depression and cardiometabolic abnormalities are likely complex due to the possibility of bidirectional associations of predictors and interactions of shared diabetes risk factors.

### Strengths and Limitations

This study had several noteworthy strengths, including a large population-based design, objectively measured cardiometabolic risk factors, and a long follow-up period allowing for an informative prospective analysis. We also included components of both metabolic syndrome and systemic inflammation, thus assessing a broad range of cardiometabolic risk factors. As well, the ELSA dataset included repeated assessments of diabetes development every 2 years over the approximately 8 year follow-up period, and allowed for the assessment of a variety of socio-demographic, lifestyle and clinical variables.

Despite its strengths, this study also had several limitations. There is a possibility of reporting bias as incidence of diabetes and date of diagnosis were determined via self-report and were not validated by a clinical diagnosis. However, in general there is good agreement between self-reported and measured diabetes [52,53]. Depressive symptoms were also self-reported using the CES-D scale, which is considered a screening tool rather than a clinical diagnostic tool. Furthermore, since the CES-D scale assesses depressive symptoms experienced within the past week, transient distress may have been captured by this assessment rather than clinical depression. However, the CES-D scale is widely used to identify individuals at high risk of depression [24]. Only older adults were included in the analysis, thus it is unclear if these results can be generalized to a population of younger adults. According to the Centres for Disease Control and Prevention, the mean age of diagnosis of diabetes is around 54 years [54], and given that the average age of our sample population was around 64 years, a significant proportion of the population had already developed diabetes and was thus excluded. No information on diet, an important health related variable, was available for assessment at baseline [55]. Because the sample consisted of older adults, survival bias is a concern. Only those who survived long enough to develop diabetes were identified as new cases and thus accounted for in the analysis. This may lead to an underestimation of the relationship between depressive symptoms, cardiometabolic abnormalities and diabetes incidence, as it is possible that more potential cases of diabetes could have been detected had the participants lived long enough. Attrition is another concern of prospective national cohort studies such as ELSA. If those who were lost to follow-up differed from those who remained in the study with respect to depression, cardiometabolic abnormalities or diabetes, bias may have been introduced. Lastly, application of these results to other ethnic groups may be limited since the majority of the sample was Caucasian (98%).

### Implications

Our findings show that older adults with co-occurring high depressive symptoms and cardiometabolic abnormalities are at an increased risk of developing diabetes even after adjusting for socio-demographic, lifestyle and clinical variables. In our study, among those with high depressive symptoms, over one third also suffered from cardiometabolic abnormalities. Those with co-occurring high depressive symptoms and cardiometabolic abnormalities may be a particularly important group as they suffer from both mental and somatic health issues as well as are at an increased risk of additional lifestyle and health-related factors associated with poor health outcomes. Screening primary care patients not only for depression but also for cardiometabolic risk factors and delivering necessary interventions may play an important role in preventing diabetes development.

Future research on chronic disease should consider other psychiatric assessments such as structured clinical interviews as well as measurements of other biological markers of cardiometabolic risk, to help elucidate the mechanisms that underlie the intricate associations between depression, cardiometabolic abnormalities and diabetes. In addition, it would be important to study these associations in younger cohorts with an earlier onset of type 2 diabetes. This research could have important clinical and public health implications including prevention and treatment of these conditions.

## Conclusion

Our study suggests that those with co-occurring high depressive symptoms and cardiometabolic abnormalities have an increased risk of developing type 2 diabetes after adjusting for socio-demographic, lifestyle and clinical variables.

## Supporting Information

S1 Table Life table analysis of the four depressive symptom and cardiometabolic abnormality groupings. (DOCX)

S2 Table Sensitivity analyses using complete-case analyses. (DOCX)

S3 Table Sensitivity analyses using cutoff of ≥3 depressive symptoms. (DOCX)

S4 Table Sensitivity analyses using cutoff of ≥5 depressive symptoms. (DOCX)

S5 Table Sensitivity analyses using cutoff of ≥2 cardiometabolic risk factors. (DOCX)

S6 Table Sensitivity analyses using cutoff of ≥4 cardiometabolic risk factors. (DOCX)

S7 Table Sensitivity analyses including those with undiagnosed diabetes (HbA1c ≥6.5% or ≥48 mmol/mol) in the analysis sample. (DOCX)
