# Supplementary material for: Risk of Diabetes in Older Adults with Co-Occurring Depressive Symptoms and Cardiometabolic Abnormalities: Prospective Analysis from the English Longitudinal Study of Ageing
Source: PLoS One. 2016 May 26;11(5):e0155741. doi: 10.1371/journal.pone.0155741 (PMC4882076; doi:10.1371/journal.pone.0155741)
Supplement: S1 Table — (DOCX) [file pone.0155741.s001.docx]

**S1 Table: Life Table analysis of the four depressive symptom and cardiometabolic abnormality groupings.**

The four tables below demonstrate the number of people at risk for each group at each time period of analysis.

**S1 Table A: Group DCM (comorbid high depressive symptoms and cardiometabolic abnormalities group) (N=219)**

Beg.

Interval^a^ Total^b^ Event^c^ Lost^d^ Survival^e^

--------------------------------------------------

13 14 219 1 0 0.9954

19 20 218 0 1 0.9954

21 22 217 0 7 0.9954

22 23 210 0 2 0.9954

23 24 208 1 2 0.9906

24 25 205 0 2 0.9906

25 26 203 0 1 0.9906

29 30 202 0 1 0.9906

30 31 201 1 0 0.9857

31 32 200 1 0 0.9808

33 34 199 1 0 0.9758

34 35 198 1 0 0.9709

39 40 197 1 0 0.9660

41 42 196 1 0 0.9611

42 43 195 0 1 0.9611

43 44 194 0 1 0.9611

44 45 193 3 0 0.9461

45 46 190 0 2 0.9461

46 47 188 0 2 0.9461

47 48 186 0 1 0.9461

48 49 185 0 1 0.9461

49 50 184 0 2 0.9461

50 51 182 1 1 0.9409

51 52 180 1 1 0.9357

52 53 178 1 0 0.9304

53 54 177 1 1 0.9251

54 55 175 1 1 0.9198

55 56 173 0 1 0.9198

57 58 172 3 0 0.9038

63 64 169 1 0 0.8984

64 65 168 1 0 0.8931

65 66 167 1 0 0.8877

66 67 166 3 0 0.8717

67 68 163 0 1 0.8717

68 69 162 1 2 0.8663

69 70 159 0 1 0.8663

70 71 158 0 2 0.8663

71 72 156 0 2 0.8663

72 73 154 1 3 0.8606

73 74 150 0 2 0.8606

75 76 148 0 2 0.8606

76 77 146 0 1 0.8606

86 87 145 1 0 0.8547

87 88 144 0 2 0.8547

88 89 142 0 4 0.8547

89 90 138 0 1 0.8547

90 91 137 0 10 0.8547

91 92 127 0 12 0.8547

92 93 115 0 13 0.8547

93 94 102 0 28 0.8547

94 95 74 1 12 0.8421

95 96 61 0 19 0.8421

96 97 42 0 12 0.8421

97 98 30 0 12 0.8421

98 99 18 0 10 0.8421

99 100 8 0 5 0.8421

100 101 3 0 2 0.8421

106 107 1 0 1 0.8421

^a^Interval: The time period (in months) during which an event (diagnosis of type 2 diabetes) occurred or the period during which participants were lost to follow-up.

^b^Beg.Total: The total number of participants at the beginning of the time interval.

^c^Event: The number of participants diagnosed with type 2 diabetes at during the time interval.

^d^Lost: The number of participants lost to follow-up during the time interval.

^e^Survival: Cumulative survival probability of the group at the time interval.

**S1 Table B: Group DnoCM (high depressive symptoms only group) (N=338)**

Beg.

Interval^a^ Total^b^ Event^c^ Lost^d^ Survival^e^

--------------------------------------------------

11 12 338 2 0 0.9941

18 19 336 0 3 0.9941

19 20 333 0 3 0.9941

20 21 330 0 6 0.9941

21 22 324 0 8 0.9941

22 23 316 0 5 0.9941

23 24 311 0 5 0.9941

24 25 306 0 2 0.9941

26 27 304 0 2 0.9941

29 30 302 0 3 0.9941

36 37 299 1 0 0.9908

41 42 298 0 2 0.9908

42 43 296 1 1 0.9874

43 44 294 0 1 0.9874

44 45 293 0 1 0.9874

46 47 292 0 4 0.9874

47 48 288 0 5 0.9874

48 49 283 0 2 0.9874

49 50 281 0 2 0.9874

51 52 279 0 2 0.9874

52 53 277 0 4 0.9874

53 54 273 0 2 0.9874

54 55 271 0 1 0.9874

55 56 270 0 1 0.9874

60 61 269 2 0 0.9801

66 67 267 0 1 0.9801

67 68 266 1 2 0.9764

68 69 263 0 3 0.9764

69 70 260 0 1 0.9764

70 71 259 0 4 0.9764

71 72 255 1 2 0.9725

72 73 252 0 8 0.9725

73 74 244 0 4 0.9725

74 75 240 0 1 0.9725

75 76 239 0 2 0.9725

76 77 237 0 2 0.9725

78 79 235 0 1 0.9725

79 80 234 0 1 0.9725

80 81 233 1 0 0.9683

85 86 232 0 1 0.9683

86 87 231 0 1 0.9683

87 88 230 0 3 0.9683

88 89 227 0 4 0.9683

89 90 223 0 9 0.9683

90 91 214 0 7 0.9683

91 92 207 0 25 0.9683

92 93 182 0 25 0.9683

93 94 157 0 39 0.9683

94 95 118 0 30 0.9683

95 96 88 0 37 0.9683

96 97 51 0 20 0.9683

97 98 31 0 9 0.9683

98 99 22 0 7 0.9683

99 100 15 0 7 0.9683

100 101 8 0 4 0.9683

101 102 4 0 1 0.9683

102 103 3 0 1 0.9683

103 104 2 0 2 0.9683

^a^Interval: The time period (in months) during which an event (diagnosis of type 2 diabetes) occurred or the period during which participants were lost to follow-up.

^b^Beg.Total: The total number of participants at the beginning of the time interval.

^c^Event: The number of participants diagnosed with type 2 diabetes at during the time interval.

^d^Lost: The number of participants lost to follow-up during the time interval.

^e^Survival: Cumulative survival probability of the group at the time interval.

**S1 Table C: Group noDCM (cardiometabolic abnormalities only group) (N=1180)**

Beg.

Interval^a^ Total^b^ Event^c^ Lost^d^ Survival^e^

--------------------------------------------------

2 3 1180 1 0 0.9992

4 5 1179 1 0 0.9983

6 7 1178 2 0 0.9966

7 8 1176 1 0 0.9958

8 9 1175 1 0 0.9949

10 11 1174 2 0 0.9932

11 12 1172 2 0 0.9915

12 13 1170 1 0 0.9907

13 14 1169 1 0 0.9898

14 15 1168 1 0 0.9890

16 17 1167 0 2 0.9890

17 18 1165 2 3 0.9873

18 19 1160 1 1 0.9864

19 20 1158 0 4 0.9864

20 21 1154 0 13 0.9864

21 22 1141 3 21 0.9838

22 23 1117 1 11 0.9829

23 24 1105 1 17 0.9820

24 25 1087 2 12 0.9802

25 26 1073 0 4 0.9802

26 27 1069 0 2 0.9802

27 28 1067 1 1 0.9793

28 29 1065 0 1 0.9793

29 30 1064 1 1 0.9784

30 31 1062 1 0 0.9775

32 33 1061 1 0 0.9765

33 34 1060 2 0 0.9747

34 35 1058 2 1 0.9728

35 36 1055 4 0 0.9692

36 37 1051 1 0 0.9682

37 38 1050 1 0 0.9673

38 39 1049 4 0 0.9636

39 40 1045 1 0 0.9627

40 41 1044 4 1 0.9590

41 42 1039 1 5 0.9581

42 43 1033 1 3 0.9572

43 44 1029 1 4 0.9562

44 45 1024 1 4 0.9553

45 46 1019 1 7 0.9544

46 47 1011 0 9 0.9544

47 48 1002 4 8 0.9505

48 49 990 0 15 0.9505

49 50 975 0 5 0.9505

50 51 970 0 5 0.9505

51 52 965 1 6 0.9495

52 53 958 0 7 0.9495

53 54 951 0 1 0.9495

54 55 950 3 1 0.9465

55 56 946 1 2 0.9455

56 57 943 1 0 0.9445

58 59 942 1 0 0.9435

59 60 941 2 0 0.9415

60 61 939 2 0 0.9395

62 63 937 1 0 0.9385

63 64 936 3 0 0.9355

64 65 933 1 2 0.9345

65 66 930 0 2 0.9345

66 67 928 1 0 0.9335

67 68 927 2 4 0.9315

68 69 921 3 7 0.9284

69 70 911 2 7 0.9264

70 71 902 1 6 0.9254

71 72 895 2 17 0.9233

72 73 876 0 12 0.9233

73 74 864 0 9 0.9233

74 75 855 0 5 0.9233

75 76 850 0 2 0.9233

76 77 848 1 3 0.9222

77 78 844 0 1 0.9222

78 79 843 1 0 0.9211

80 81 842 1 0 0.9200

81 82 841 1 0 0.9189

82 83 840 1 0 0.9178

83 84 839 1 0 0.9167

84 85 838 1 0 0.9156

85 86 837 0 1 0.9156

86 87 836 2 2 0.9134

87 88 832 1 16 0.9123

88 89 815 2 8 0.9101

89 90 805 1 22 0.9089

90 91 782 1 57 0.9077

91 92 724 1 64 0.9064

92 93 659 2 80 0.9035

93 94 577 1 120 0.9017

94 95 456 0 114 0.9017

95 96 342 0 90 0.9017

96 97 252 0 82 0.9017

97 98 170 0 75 0.9017

98 99 95 0 37 0.9017

99 100 58 0 32 0.9017

100 101 26 0 8 0.9017

101 102 18 0 10 0.9017

102 103 8 0 6 0.9017

104 105 2 0 1 0.9017

105 106 1 0 1 0.9017

^a^Interval: The time period (in months) during which an event (diagnosis of type 2 diabetes) occurred or the period during which participants were lost to follow-up.

^b^Beg.Total: The total number of participants at the beginning of the time interval.

^c^Event: The number of participants diagnosed with type 2 diabetes at during the time interval.

^d^Lost: The number of participants lost to follow-up during the time interval.

^e^Survival: Cumulative survival probability of the group at the time interval.

**S1 Table D: Group noDnoCM (no or low depressive symptoms and no cardiometabolic abnormalities group) (N=2717)**

Beg.

Interval^a^ Total^b^ Event^c^ Lost^d^ Survival^e^

--------------------------------------------------

1 2 2717 1 0 0.9996

3 4 2716 1 0 0.9993

7 8 2715 1 0 0.9989

8 9 2714 1 0 0.9985

11 12 2713 1 0 0.9982

15 16 2712 1 1 0.9978

16 17 2710 0 2 0.9978

17 18 2708 0 2 0.9978

18 19 2706 0 13 0.9978

19 20 2693 0 26 0.9978

20 21 2667 0 29 0.9978

21 22 2638 0 36 0.9978

22 23 2602 1 36 0.9974

23 24 2565 1 25 0.9970

24 25 2539 0 16 0.9970

25 26 2523 0 9 0.9970

26 27 2514 0 7 0.9970

27 28 2507 0 3 0.9970

28 29 2504 0 5 0.9970

29 30 2499 0 1 0.9970

31 32 2498 0 1 0.9970

32 33 2497 1 1 0.9966

33 34 2495 1 0 0.9962

34 35 2494 3 2 0.9950

35 36 2489 1 0 0.9946

36 37 2488 1 0 0.9942

37 38 2487 0 1 0.9942

39 40 2486 0 3 0.9942

40 41 2483 0 4 0.9942

41 42 2479 0 7 0.9942

42 43 2472 0 7 0.9942

43 44 2465 1 11 0.9938

44 45 2453 0 9 0.9938

45 46 2444 0 18 0.9938

46 47 2426 1 19 0.9934

47 48 2406 0 18 0.9934

48 49 2388 3 22 0.9921

49 50 2363 0 16 0.9921

50 51 2347 0 7 0.9921

51 52 2340 1 3 0.9917

52 53 2336 0 8 0.9917

53 54 2328 1 6 0.9913

54 55 2321 0 4 0.9913

55 56 2317 1 4 0.9909

56 57 2312 2 2 0.9900

57 58 2308 1 0 0.9896

58 59 2307 0 1 0.9896

59 60 2306 1 0 0.9892

60 61 2305 1 0 0.9887

61 62 2304 1 0 0.9883

63 64 2303 1 0 0.9879

64 65 2302 1 2 0.9874

65 66 2299 1 1 0.9870

66 67 2297 1 3 0.9866

67 68 2293 0 6 0.9866

68 69 2287 1 16 0.9861

69 70 2270 1 16 0.9857

70 71 2253 1 23 0.9853

71 72 2229 1 23 0.9848

72 73 2205 1 23 0.9844

73 74 2181 1 20 0.9839

74 75 2160 0 11 0.9839

75 76 2149 0 7 0.9839

76 77 2142 0 4 0.9839

77 78 2138 1 6 0.9835

78 79 2131 0 5 0.9835

79 80 2126 1 1 0.9830

82 83 2124 3 0 0.9816

83 84 2121 2 0 0.9807

84 85 2119 2 1 0.9798

85 86 2116 1 4 0.9793

86 87 2111 1 6 0.9788

87 88 2104 0 19 0.9788

88 89 2085 2 42 0.9779

89 90 2041 0 59 0.9779

90 91 1982 2 110 0.9769

91 92 1870 1 149 0.9763

92 93 1720 0 234 0.9763

93 94 1486 0 275 0.9763

94 95 1211 0 307 0.9763

95 96 904 0 239 0.9763

96 97 665 0 236 0.9763

97 98 429 0 191 0.9763

98 99 238 0 96 0.9763

99 100 142 0 67 0.9763

100 101 75 0 38 0.9763

101 102 37 0 21 0.9763

102 103 16 0 10 0.9763

103 104 6 0 4 0.9763

104 105 2 0 1 0.9763

106 107 1 0 1 0.9763

^a^Interval: The time period (in months) during which an event (diagnosis of type 2 diabetes) occurred or the period during which participants were lost to follow-up.

^b^Beg.Total: The total number of participants at the beginning of the time interval.

^c^Event: The number of participants diagnosed with type 2 diabetes at during the time interval.

^d^Lost: The number of participants lost to follow-up during the time interval.

^e^Survival: Cumulative survival probability of the group at the time interval.
