# Supplementary material for: Risk of Diabetes in Older Adults with Co-Occurring Depressive Symptoms and Cardiometabolic Abnormalities: Prospective Analysis from the English Longitudinal Study of Ageing
Source: PLoS One. 2016 May 26;11(5):e0155741. doi: 10.1371/journal.pone.0155741 (PMC4882076; doi:10.1371/journal.pone.0155741)
Supplement: S2 Table — (DOCX) [file pone.0155741.s002.docx]

**S2 Table. Sensitivity analyses using complete-case analyses.**

| Cox Regression HRs (95% CI) | noDnoCM | noDCM | DnoCM | DCM | S (95% CI) |
| --- | --- | --- | --- | --- | --- |
| Model 1: Unadjusted | 1.00 | 4.41 (3.18, 6.13) | 1.40 (0.69, 2.83) | 7.03 (4.48, 11.02) | 1.58 (0.85, 2.73) |
| Model 2: Adjusted for age, sex, education, income | 1.00 | 4.17 (2.98, 5.83) | 1.38 (0.68, 2.82) | 6.01 (3.76, 9.61) | 1.41 (0.75, 2.53) |
| Model 3: Model 2 + adjusted for physical activity, smoking, alcohol consumption | 1.00 | 4.31 (3.02, 6.16) | 1.44 (0.68, 3.08) | 7.00 (4.24, 11.55) | 1.60 (0.84, 2.92) |
| Model 4: Model 3 + adjusted for cardiovascular comorbidity | 1.00 | 4.06 (2.84, 5.82) | 1.42 (0.66, 3.03) | 6.48 (3.92, 10.72) | 1.57 (0.82, 3.00) |

DCM: comorbid high depressive symptoms and cardiometabolic abnormalities group

DnoCM: high depressive symptoms only group

noDCM: cardiometabolic abnormalities only group

noDnoCM: no or low depressive symptoms and no cardiometabolic abnormalities group

S: Synergy Index

HR: Hazard Ratio

CI: Confidence Interval
