# Supplementary material for: Risk of Diabetes in Older Adults with Co-Occurring Depressive Symptoms and Cardiometabolic Abnormalities: Prospective Analysis from the English Longitudinal Study of Ageing
Source: PLoS One. 2016 May 26;11(5):e0155741. doi: 10.1371/journal.pone.0155741 (PMC4882076; doi:10.1371/journal.pone.0155741)
Supplement: S3 Table — (DOCX) [file pone.0155741.s003.docx]

**S3 Table. Sensitivity analyses using cutoff of ≥3 depressive symptoms.**

| Cox Regression HRs (95% CI) | noDnoCM | noDCM | DnoCM | DCM |
| --- | --- | --- | --- | --- |
| Model 1: Unadjusted | 1.00 | 4.22 (3.00, 5.94) | 1.14 (0.61, 2.14) | 6.49 (4.30, 9.80) |
| Model 2: Adjusted for age, sex, education, income | 1.00 | 3.97 (2.80, 5.62) | 1.08 (0.57, 2.05) | 5.62 (3.65, 8.65) |
| Model 3: Model 2 + adjusted for physical activity, smoking, alcohol consumption | 1.00 | 4.16 (2.86, 6.03) | 1.18 (0.60, 2.30) | 6.27 (3.94, 9.96) |
| Model 4: Model 3 + adjusted for cardiovascular comorbidity | 1.00 | 3.91 (2.69, 5.69) | 1.14 (0.58, 2.23) | 5.76 (3.61, 9.19) |

DCM: comorbid high depressive symptoms and cardiometabolic abnormalities group

DnoCM: high depressive symptoms only group

noDCM: cardiometabolic abnormalities only group

noDnoCM: no or low depressive symptoms and no cardiometabolic abnormalities group

HR: Hazard Ratio

CI: Confidence Interval
