# Supplementary material for: Risk of Diabetes in Older Adults with Co-Occurring Depressive Symptoms and Cardiometabolic Abnormalities: Prospective Analysis from the English Longitudinal Study of Ageing
Source: PLoS One. 2016 May 26;11(5):e0155741. doi: 10.1371/journal.pone.0155741 (PMC4882076; doi:10.1371/journal.pone.0155741)
Supplement: S4 Table — (DOCX) [file pone.0155741.s004.docx]

**S4 Table. Sensitivity analyses using cutoff of ≥5 depressive symptoms.**

| Cox Regression HRs (95% CI) | noDnoCM | noDCM | DnoCM | DCM |
| --- | --- | --- | --- | --- |
| Model 1: Unadjusted | 1.00 | 4.18 (3.05, 5.72) | 0.65 (0.20, 2.08) | 7.12 (4.41, 11.51) |
| Model 2: Adjusted for age, sex, education, income | 1.00 | 3.93 (2.85, 5.41) | 0.64 (0.20, 2.05) | 5.92 (3.59, 9.78) |
| Model 3: Model 2 + adjusted for physical activity, smoking, alcohol consumption | 1.00 | 4.11 (2.92, 5.79) | 0.76 (0.24, 2.44) | 6.74 (3.93, 11.54) |
| Model 4: Model 3 + adjusted for cardiovascular comorbidity | 1.00 | 3.86 (2.73, 5.44) | 0.74 (0.23, 2.37) | 6.32 (3.69, 10.85) |

DCM: comorbid high depressive symptoms and cardiometabolic abnormalities group

DnoCM: high depressive symptoms only group

noDCM: cardiometabolic abnormalities only group

noDnoCM: no or low depressive symptoms and no cardiometabolic abnormalities group

HR: Hazard Ratio

CI: Confidence Interval
