# Supplementary material for: Risk of Diabetes in Older Adults with Co-Occurring Depressive Symptoms and Cardiometabolic Abnormalities: Prospective Analysis from the English Longitudinal Study of Ageing
Source: PLoS One. 2016 May 26;11(5):e0155741. doi: 10.1371/journal.pone.0155741 (PMC4882076; doi:10.1371/journal.pone.0155741)
Supplement: S5 Table — (DOCX) [file pone.0155741.s005.docx]

**S5 Table. Sensitivity analyses using cutoff of ≥2 cardiometabolic risk factors.**

| Cox Regression HRs (95% CI) | noDnoCM | noDCM | DnoCM | DCM |
| --- | --- | --- | --- | --- |
| Model 1: Unadjusted | 1.00 | 5.93 (3.63, 9.70) | 1.46 (0.43, 4.94) | 9.93 (5.62, 17.53) |
| Model 2: Adjusted for age, sex, education, income | 1.00 | 5.24 (3.19, 8.59) | 1.37 (0.40, 4.68) | 8.09 (4.52, 14.48) |
| Model 3: Model 2 + adjusted for physical activity, smoking, alcohol consumption | 1.00 | 5.36 (3.17, 9.08) | 1.13 (0.26, 4.93) | 9.09 (4.88, 16.95) |
| Model 4: Model 3 + adjusted for cardiovascular comorbidity | 1.00 | 5.09 (3.00, 8.61) | 1.13 (0.26, 4.95) | 8.37 (4.48, 15.63) |

DCM: comorbid high depressive symptoms and cardiometabolic abnormalities group

DnoCM: high depressive symptoms only group

noDCM: cardiometabolic abnormalities only group

noDnoCM: no or low depressive symptoms and no cardiometabolic abnormalities group

HR: Hazard Ratio

CI: Confidence Interval
