# Supplementary material for: Risk of Diabetes in Older Adults with Co-Occurring Depressive Symptoms and Cardiometabolic Abnormalities: Prospective Analysis from the English Longitudinal Study of Ageing
Source: PLoS One. 2016 May 26;11(5):e0155741. doi: 10.1371/journal.pone.0155741 (PMC4882076; doi:10.1371/journal.pone.0155741)
Supplement: S6 Table — (DOCX) [file pone.0155741.s006.docx]

**S6 Table. Sensitivity analyses using cutoff of ≥4 cardiometabolic risk factors.**

| Cox Regression HRs (95% CI) | noDnoCM | noDCM | DnoCM | DCM |
| --- | --- | --- | --- | --- |
| Model 1: Unadjusted | 1.00 | 5.83 (4.22, 8.05) | 1.83 (1.15, 2.91) | 6.94 (4.08, 11.78) |
| Model 2: Adjusted for age, sex, education, income | 1.00 | 5.54 (3.98, 7.70) | 1.81 (1.13, 2.90) | 5.51 (3.16, 9.62) |
| Model 3: Model 2 + adjusted for physical activity, smoking, alcohol consumption | 1.00 | 5.51 (3.87, 7.86) | 2.02 (1.23, 3.30) | 5.88 (3.21, 10.76) |
| Model 4: Model 3 + adjusted for cardiovascular comorbidity | 1.00 | 5.19 (3.64, 7.41) | 1.99 (1.21, 3.25) | 5.36 (2.92, 9.82) |

DCM: comorbid high depressive symptoms and cardiometabolic abnormalities group

DnoCM: high depressive symptoms only group

noDCM: cardiometabolic abnormalities only group

noDnoCM: no or low depressive symptoms and no cardiometabolic abnormalities group

HR: Hazard Ratio

CI: Confidence Interval
