# Supplementary material for: Risk of Diabetes in Older Adults with Co-Occurring Depressive Symptoms and Cardiometabolic Abnormalities: Prospective Analysis from the English Longitudinal Study of Ageing
Source: PLoS One. 2016 May 26;11(5):e0155741. doi: 10.1371/journal.pone.0155741 (PMC4882076; doi:10.1371/journal.pone.0155741)
Supplement: S7 Table — (DOCX) [file pone.0155741.s007.docx]

**S7 Table. Sensitivity analyses including those with undiagnosed diabetes (HbA1c ≥6.5% or ≥48 mmol/mol) in the analysis sample.**

| Cox Regression HRs (95% CI) | noDnoCM | noDCM | DnoCM | DCM |
| --- | --- | --- | --- | --- |
| Model 1: Unadjusted | 1.00 | 5.46 (4.04, 7.37) | 1.25 (0.62, 2.51) | 8.26 (5.52, 12.34) |
| Model 2: Adjusted for age, sex, education, income | 1.00 | 5.17 (3.81, 7.02) | 1.25 (0.62, 2.52) | 7.15 (4.71, 10.85) |
| Model 3: Model 2 + adjusted for physical activity, smoking, alcohol consumption | 1.00 | 5.33 (3.84, 7.38) | 1.28 (0.60, 2.72) | 7.92 (5.04, 12.45) |
| Model 4: Model 3 + adjusted for cardiovascular comorbidity | 1.00 | 5.14 (3.70, 7.14) | 1.27 (0.60, 2.69) | 7.55 (4.80, 11.89) |

DCM: comorbid high depressive symptoms and cardiometabolic abnormalities group

DnoCM: high depressive symptoms only group

noDCM: cardiometabolic abnormalities only group

noDnoCM: no or low depressive symptoms and no cardiometabolic abnormalities group

HR: Hazard Ratio

CI: Confidence Interval
